# Human adipose tissue-derived mesenchymal stem cells alleviate atopic dermatitis via regulation of B lymphocyte maturation

**DOI:** 10.18632/oncotarget.13473

**Published:** 2016-11-19

**Authors:** Tae-Hoon Shin, Byung-Chul Lee, Soon Won Choi, Ji-Hee Shin, Insung Kang, Jin Young Lee, Jae-Jun Kim, Hong-Ki Lee, Jae-Eon Jung, Yong-Woon Choi, Sung-Hoon Lee, Jin-Sang Yoon, Jin-Sub Choi, Chi-Seung Lee, Yoojin Seo, Hyung-Sik Kim, Kyung-Sun Kang

**Affiliations:** ^1^ Adult Stem Cell Research Center, College of Veterinary Medicine, Seoul National University, Seoul 08826, South Korea; ^2^ Research Institute for Veterinary Science, College of Veterinary Medicine, Seoul National University, Seoul 08826, South Korea; ^3^ Biotechnology Institute, EHL-BIO Co., Ltd., Uiwang 16006, South Korea; ^4^ School of Medicine, Pusan National University, Busan 49241, South Korea; ^5^ Biomedical Research Institute, Pusan National University Hospital, Busan 49241, South Korea

**Keywords:** mesenchymal stem cells, atopic dermatitis, B cell maturation, mast cell degranulation, distribution

## Abstract

Mesenchymal stem cell (MSC) has been applied for the therapy of allergic disorders due to its beneficial immunomodulatory abilities. However, the underlying mechanisms for therapeutic efficacy are reported to be diverse according to the source of cell isolation or the route of administration. We sought to investigate the safety and the efficacy of human adipose tissue-derived MSCs (hAT-MSCs) in mouse atopic dermatitis (AD) model and to determine the distribution of cells after intravenous administration. Murine AD model was established by multiple treatment of *Dermatophagoides farinae*. AD mice were intravenously infused with hAT-MSCs and monitored for clinical symptoms. The administration of hAT-MSCs reduced the gross and histological signatures of AD, as well as serum IgE level. hAT-MSCs were mostly detected in lung and heart of mice within 3 days after administration and were hardly detectable at 2 weeks. All of mice administered with hAT-MSCs survived until sacrifice and did not demonstrate any adverse events. Co-culture experiments revealed that hAT-MSCs significantly inhibited the proliferation and the maturation of B lymphocytes via cyclooxygenase (COX)-2 signaling. Moreover, mast cell (MC) degranulation was suppressed by hAT-MSC. In conclusion, the intravenous infusion of hAT-MSCs can alleviate AD through the regulation of B cell function.

## INTRODUCTION

Mesenchymal stem cells (MSCs) have been known to interact with cell types of both innate and adaptive immune systems, which results in the suppressive effect on proliferation, differentiation, and activation of immune cells including T cells, B cells, dendritic cells, and natural killer (NK) cells [1–4]. Indeed, a number of studies have reported that the immunomodulatory ability of MSCs can be usefully applied for the treatment of autoimmune and inflammation-related diseases, such as graft-versus-host-disease, inflammatory bowel disease, multiple sclerosis, sepsis, collagen-induced arthritis, and type I diabetes [5–10]. Moreover, recent studies, including our previous one have demonstrated that MSCs can ameliorate allergic diseases such as asthma, rhinitis and dermatitis [11–18]. However, the preventive or therapeutic potency and the mechanisms of action can be altered by the difference in the sources of MSCs or the administration routes [14, 19].

Atopic dermatitis (AD) is a chronic and relapsing skin disorder accompanied by xerosis, eczematous lesions and severe pruritus [20]. The pathogenesis of AD is characterized by excessive type 2 helper T cell (Th2)-mediated inflammatory responses, resulting in B lymphocyte-mediated increase in serum level of IgE [21–23]. Subsequent degranulation of mast cells (MCs) by IgE releases various inflammatory mediators, which recruit lymphocytes and eosinophils into the lesion. The treatment and management of AD is complicated. Generally, current clinical management of AD includes topical corticosteroids and systemic immunosuppressants. However, these drugs have been reported to carry the risk of side-effects [24, 25]. More recently, new biological agents have been developed for AD patients to specifically inhibit target molecules. However, most of these biologics have been reported to have limited and non-uniform efficacies, as well as uncertainty in long term safety in clinical trials [26]. Therefore, MSCs can be a promising candidate to replace current therapeutics because they can regulate multiple factors simultaneously in response to the inflammatory milieu.

Several recent studies have demonstrated that MSCs could suppress allergic responses in AD [14, 27]. In particular, our previous study revealed that the administration of human umbilical cord blood-derived MSCs (hUCB-MSCs) could ameliorate AD symptoms in mouse model through the regulation of MC degranulation [14]. In the study, the potency of regulation in serum IgE level by hUCB-MSCs was remarkably different depending on the route of administration. To provide the evidence and the reference in the field of stem cell therapeutics for allergic diseases, in the present study, we sought to investigate the therapeutic efficacy of intravenously administered hAT-MSCs in *Dermatophagoides farinae* (Df)-induced AD mouse model along with the safety, distribution and the mode of action.

## RESULTS

### Intravenous administration of hAT-MSCs reduces the symptoms of Df-induced atopic dermatitis in mice

**Figure 1:**
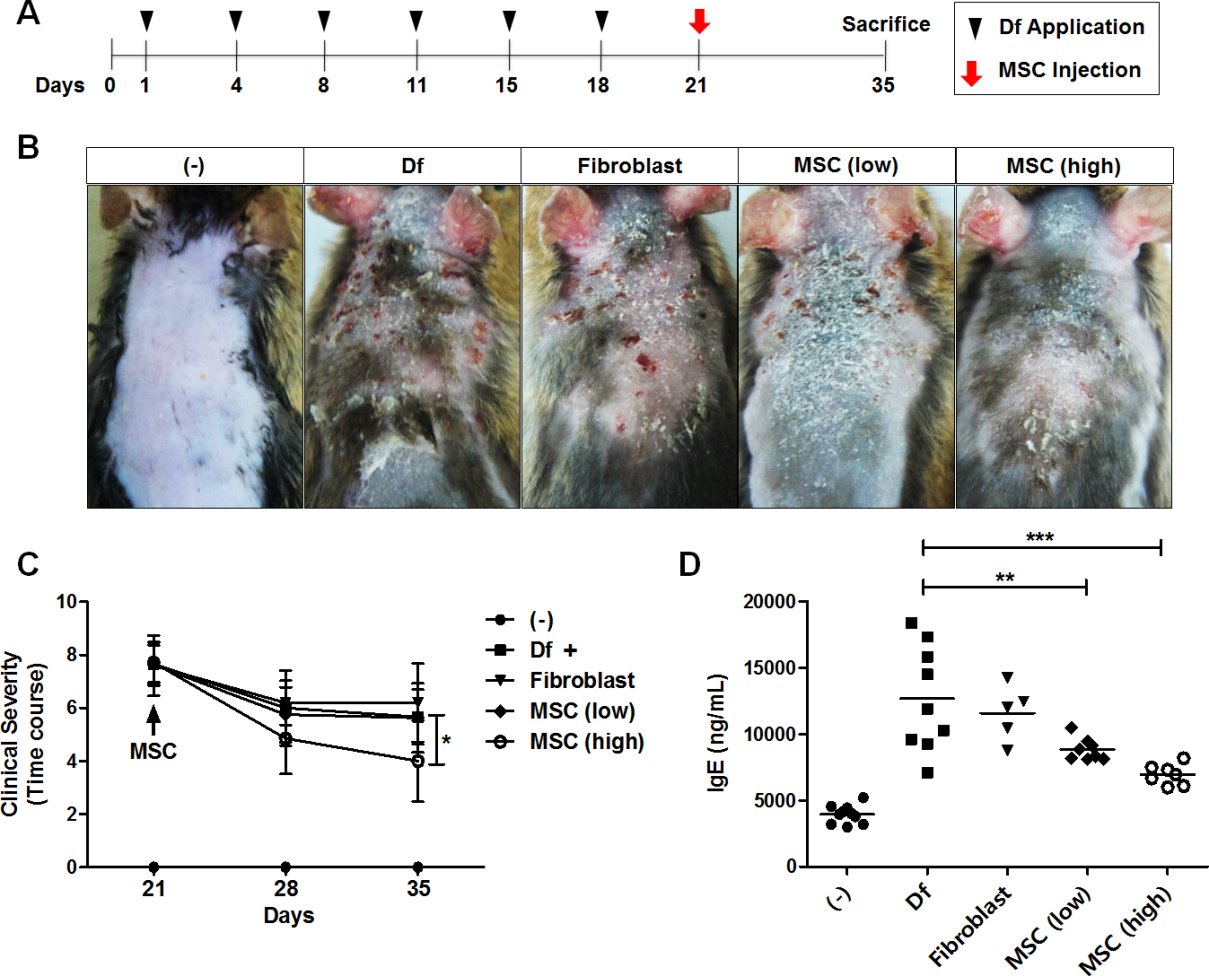
Therapeutic effect of i.v. injected hAT-MSCs in AD mice (**A**–**D**) Atopic dermatitis was induced by the repetitive application of *Dermatophagoides farinae* (Df). On day 21, after the onset of disease, two different doses of hAT-MSCs or human dermal fibroblasts were injected intravenously (i.v). (A) Scheme of AD induction and cell injection. (B) Photographs of skin gross lesions were taken for pathological evaluation. (C) Clinical severity was consistently monitored and evaluated until sacrifice. (D) On day 35, all mice were sacrificed for further analysis and serum level of IgE was measured by ELISA. Five to ten mice per group were used. *P < 0.05, **P < 0.01, ***P < 0.001. Results are shown as mean ± SD.

We first investigated whether the xenogeneic administration of hAT-MSCs could exert therapeutic effect against Df-induced murine AD. To assess the therapeutic effects, two different doses (low dose; 2 × 10^5^, high dose; 2 × 10^6^) of hAT-MSCs were injected intravenously at day 21 when AD was fully induced (Figure 1A). Human dermal fibroblasts were infused as a cell control group. None of the mice that received hAT-MSCs showed any adverse events or lethality. Interestingly, intravenous administration of high dose hAT-MSCs significantly reduced the clinical severity of AD mice, whereas low dose group did not exert effects at least in gross evaluation (Figure 1B and 1C). To determine the serum immunoglobulin level after hAT-MSC administration, serum IgE concentration was measured. The serum level of IgE was increased by AD induction and its level was significantly down-regulated by the treatment of low dose hAT-MSCs and further decreased in high dose-treated group (Figure 1D). However, fibroblast injection did not suppress serum IgE increase (Figure 1D).

**Figure 2:**
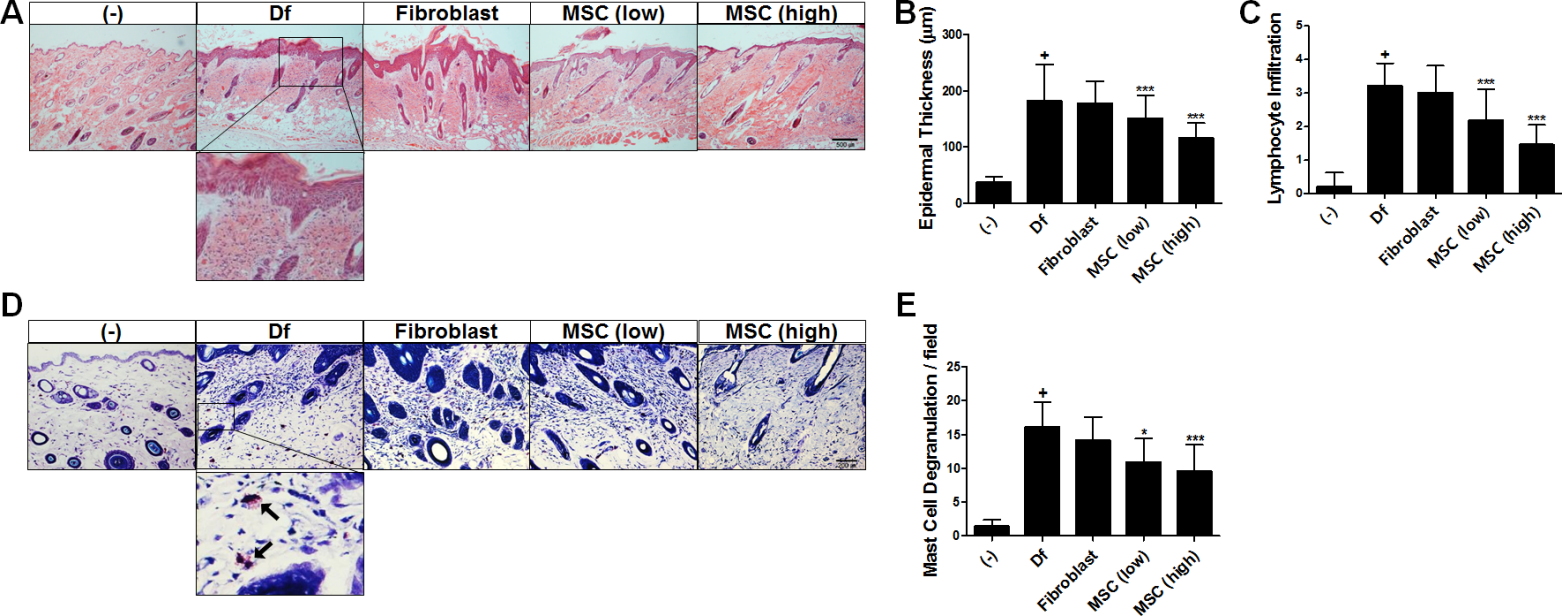
Histopathological analysis of hAT-MSC efficacy in AD mice (**A**) Paraffin-embedded sections of skin tissue from AD mice were stained with hematoxylin and eosin, scale bar = 200 μm. (**B**) Epidermal thickness and (**C**) the number of infiltrated lymphocytes were measured. (**D**) Skin sections were stained with toluidine blue, scale bar = 200 μm and (E) the number of degranulating or degranulated mast cells (indicated by arrows) was counted. Five to ten mice per group were used. *P < 0.05, **P < 0.01, ***P < 0.001. Results are shown as mean ± SD.

Histological evaluation using H&E staining revealed that the epidermal hyperplasia and lymphocyte infiltration exerted by AD induction were attenuated by hAT-MSC treatment in a dose-dependent manner (Figure 2A–2C). We next performed toluidine blue staining to determine the degranulation of MCs infiltrated in lesions. hAT-MSC administration significantly reduced the number of degranulated MCs (Figure 2D and 2E).

Taken together, our results indicate that the intravenously delivered hAT-MSCs exhibit a dose-dependent efficacy against Df-induced AD in both criteria of gross and histopathological evaluation, and that mechanisms regulating IgE production might be involved in this effect.

### Intravenously injected hAT-MSCs are mostly distributed in the lung and heart of mice and excreted within two weeks

**Figure 3:**
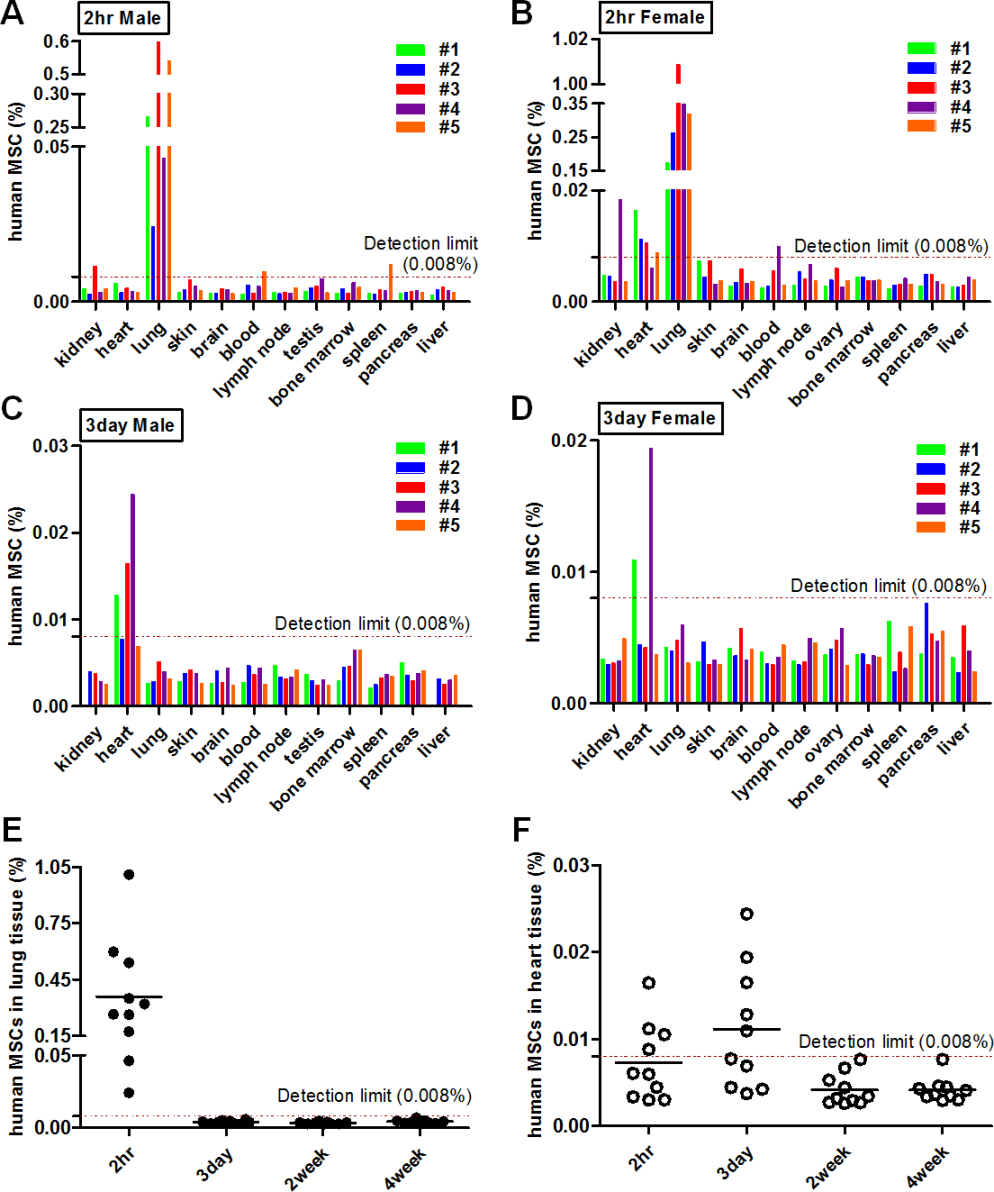
Distribution of hAT-MSCs after i.v. administration (**A**–**F**) Atopic dermatitis was induced by the application of *Dermatophagoides farinae* (Df). Mice were intravenously (i.v) administered with hAT-MSCs. At 2 hours, 3 days, 2 and 4 weeks after cell injection, mice were sacrificed and DNAs were isolated from various organs. Distribution of the delivered MSCs in AD mice was analyzed using quantitative real-time PCR analysis of the human-specific ALU gene. The concentration of human MSCs in various organs was evaluated (A–B) at 2 hours and (C–D) at 3 days after cell injection. (E–F) Quantified values of MSC distribution in major two organs, lung and heart, were integrated. Ten mice/group were used. The detection limit (0.008% of hAT-MSCs) was determined based on standard curve.

Given that the distribution of MSCs, as well as the paracrine function is crucial to elicit sufficient efficacy, we tracked and quantified the infused cells using real-time qPCR. After 2 hours of hAT-MSC administration, most of the cells (10 out of 10 mice) were detected in the lung of mice (Figure 3A, 3B and 3E). Two cases in kidney, 4 cases in heart, 2 cases in blood, and 1 case in spleen were detected among mice sacrificed at 2 hours after cell infusion (Figure 3A and 3B). At day 3 after cell injection, 5 out of 10 mice showed the cell distribution in heart and cells were hardly detectable in the other organs (Figure 3C, 3D and 3F). At week 2 and 4, hAT-MSCs were not detected in all evaluated organs of mice (Figure 3E and 3F). All forty mice administered with hAT-MSCs survived until sacrifice and did not show any adverse effects. Taken together, these findings demonstrate that intravenously delivered hAT-MSCs are mostly trapped in the lung and heart of mice followed by the excretion within a short period, implying that the therapeutic effect of i.v. infused hAT-MSCs might be the consequence of systemic inflammatory regulation rather than local action.

### hAT-MSCs suppress the proliferation and maturation of B lymphocytes via COX-2 signaling

**Figure 4:**
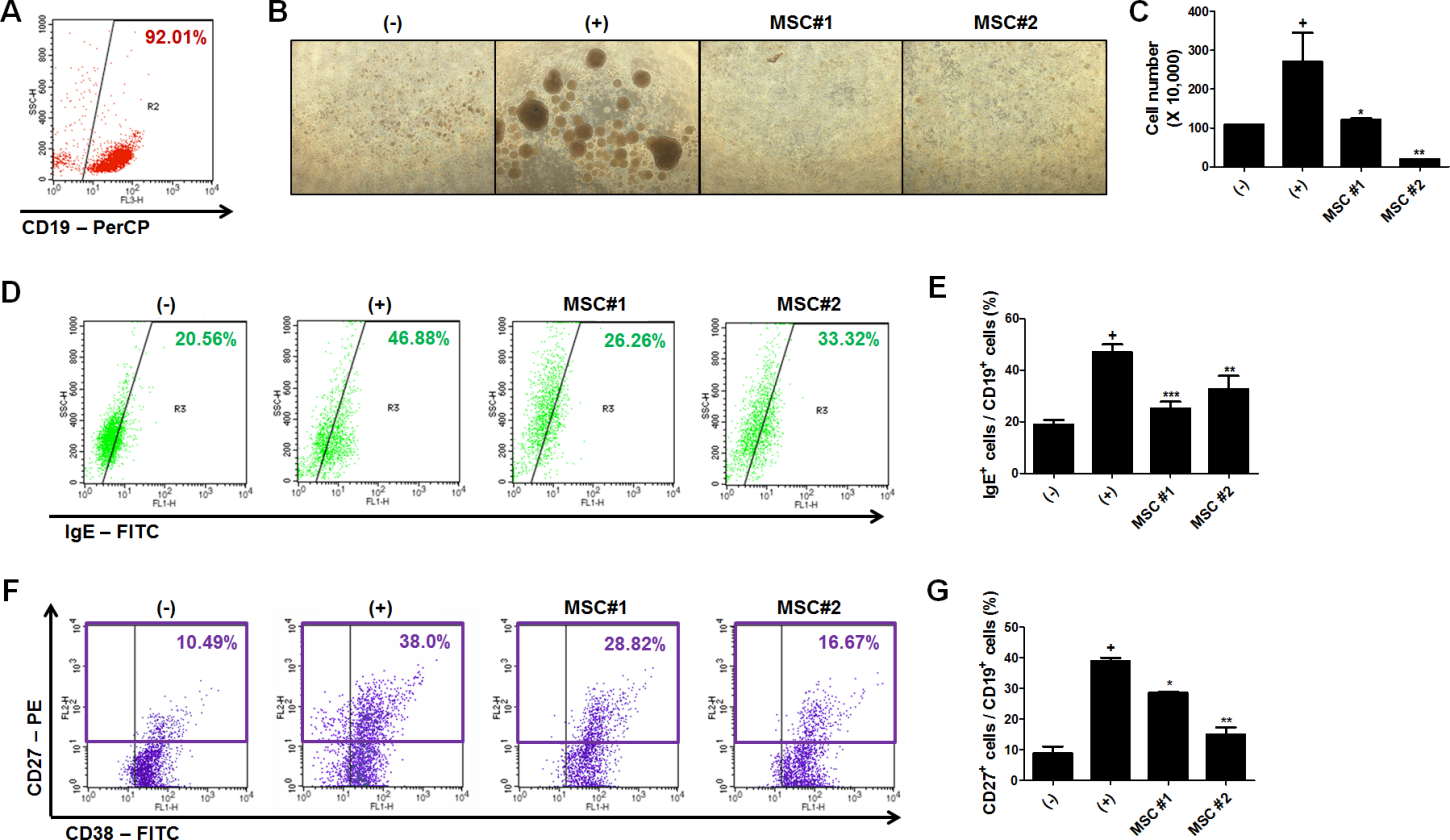
Regulation of B cell function by hAT-MSCs (**A**) Human B lymphocytes were isolated from umbilical cord blood-derived mononuclear cells and isolation purity was determined by measuring the percentage of CD19-positive cells (**B**–**G**) B lymphocytes were co-cultured with two different donor-derived hAT-MSCs after induction of maturation. The proliferation of B lymphocytes was analyzed by (B) observing phase-contrast images and (C) counting the number of CD19-positive B lymphocytes. (D–E) Intensity of IgE expression among human CD19-positive cells was confirmed by flow cytometry with or without hAT-MSCs. (F–G) Mature B lymphocyte population was determined by measuring CD27- and CD19-double positive cells using flow cytometry. *P < 0.05, **P < 0.01, ***P < 0.001. Results are 1 representative experiment of 3 or the cumulative of 3 independent experiments. Results are shown as mean ± SD.

Since we have found that hAT-MSCs could decrease the level of serum IgE in AD and this efficacy might result from the systemic regulation of allergic responses, we further investigated whether AT-MSCs could inhibit the allergic responses of B lymphocyte *in vitro*. We isolated B cells from cord blood-derived mononuclear cells and confirmed its purity before conducting subsequent experiments (Figure 4A). hAT-MSCs were added to matured B cells and co-cultured for 5 days within the range of 1:100 to 1:1 based on the MSC : B cell ratio. MSCs exhibited the maximum suppressive capability at a ratio of 1:10 (data not shown). The proliferation of B cells was significantly inhibited by co-culture with two different donor-derived hAT-MSCs (Figure 4B and 4C). Moreover, the proportion of B cells expressing IgE was significantly down-regulated by hAT-MSC addition (Figure 4D and 4E). Consistently, the proportion of mature B cells was confirmed by analyzing the surface expression of CD27 and CD38 expression, and the number of CD27^+^ B cells was decreased when hAT-MSCs were co-cultured (Figure 4F and 4G).

**Figure 5:**
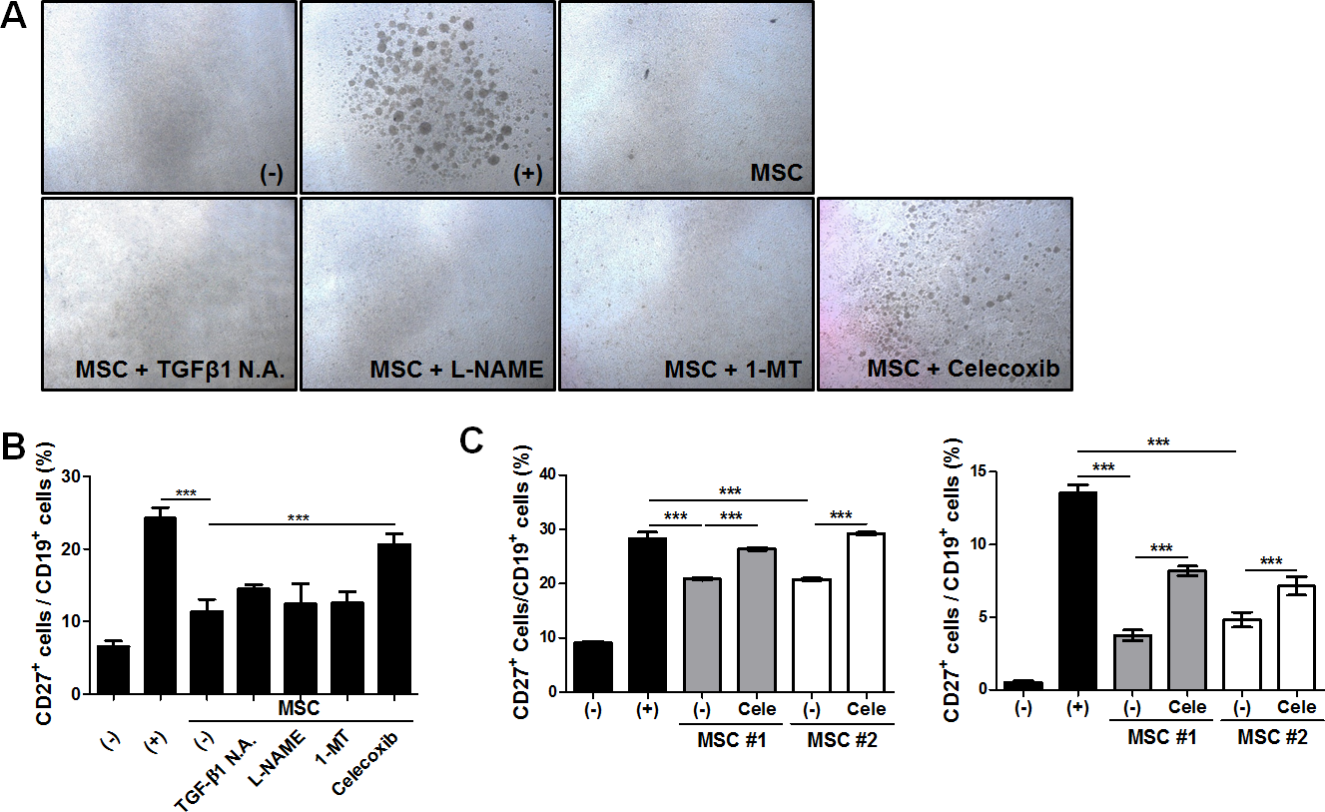
COX-2 signaling-mediated suppressive effect of hAT-MSCs on B cell function (**A**–**B**) B lymphocytes were co-cultured with hAT-MSCs after inhibition of major immunomodulatory factors including TGF-β1, iNOS, IDO-1 and COX-2. The suppressive effect of inhibitor pretreated MSCs on B cell proliferation and maturation was evaluated. (A) B cell proliferation was determined by microscopic observation. Representative phase-contrast images showing B cell proliferation. (B) CD27 expression was analyzed among the CD19^+^ B cell fraction to determine B cell maturation by flow cytometry. (**C**) MSCs or COX-2 inhibited MSCs from different donors were added to different cord blood-derived B lymphocytes and the suppressive effect of MSCs on B cell maturation was confirmed by flow cytometric analysis. ***P < 0.001. Results are 1 representative experiment of 3 or the cumulative of 3 independent experiments. Results are shown as mean ± SD.

To explore the pivotal factors responsible for the suppressive effect of hAT-MSCs on B cell functions, we inhibited the synthesis or the action of crucial factors using selective inhibitors. The crucial molecules or signals such as transforming growth factor (TGF)-β1, nitric oxide (NO), indoleamine-2, 3-dioxygenase-1 (IDO-1), and cyclooxygenase-2 (COX-2) were down-regulated with TGF-β1 neutralizing antibody, L-nitro-arginine methyl ester (L-NAME), 1-methyl tryptophan (MT) and celecoxib, respectively. Surprisingly, only the inhibition of COX-2 signaling by celecoxib significantly restored the inhibitory effects of hAT-MSCs on B cell proliferation and maturation (Figure 5A and 5B). The role of COX-2 signaling in hAT-MSCs was further confirmed by additional co-culture experiments using different cord blood-derived B cells and different adipose tissue-derived MSCs (Figure 5C). Taken together, our results support the model that systemic delivery of hAT-MSCs can suppress B cell maturation to suppress the synthesis of IgE by matured B cells.

### hAT-MSCs inhibit MC degranulation through the concerted action of TGF-β1 and COX-2 signaling

**Figure 6:**
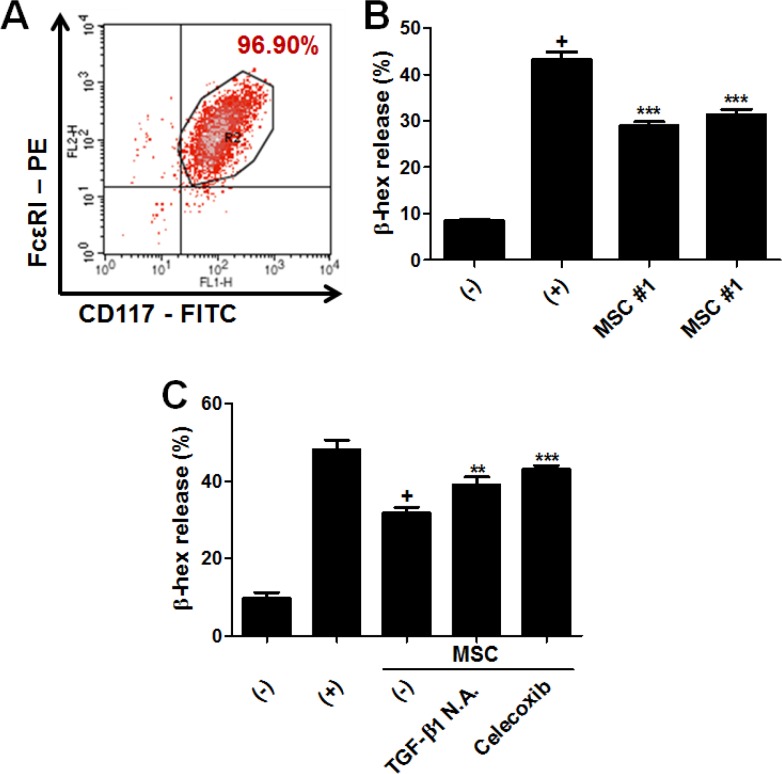
hAT-MSCs suppress MC degranulation via TGF-β1 production and COX-2 signaling (**A**) LAD-2 cells, the human mast cell line, were characterized by detecting the expression of surface markers specific for mast cells including CD117 (c-kit) and FcεRI. (**B**) LAD-2 cells were co-cultured with two different donor-derived hAT-MSCs, using transwell system and degranulation was assessed by measuring β-hexaminidase release. (**C**) TGF- β1- and COX-2-inhibited MSCs were added to LAD-2 cells and β-hex release was detected. **P < 0.01, ***P < 0.001. Results are 1 representative experiment of 3 or the cumulative of 3 independent experiments. Results are shown as mean ± SD.

In our previous study, we reported that the suppression of MC degranulation by hUCB-MSCs is mediated by paracrine factors and their upstream signaling [14]. Therefore, we investigated whether this inhibitory effect of MSCs on MCs is reproducible in this study. LAD-2 cells, human mast cell line, were periodically characterized by detecting specific surface markers and further used for degranulation assay (Figure 6A). LAD-2 cells were co-cultured with hAT-MSCs within the range of 1:10 to 10:1 based on the MSC : LAD-2 cell ratio during the induction of degranulation. The secretion of granules from LAD-2 cells was assessed by measuring β-hexosaminidase (β-hex) release. hAT-MSCs exhibited the maximum inhibitory effect at a ratio of 1:1 (data not shown). As expected, hAT-MSCs exerted the suppressive effect on LAD-2 cell degranulation (Figure 6B). Furthermore, inhibition of TGF-β1 as well as COX-2 resulted in the partial loss of this effect (Figure 6C).

## DISCUSSION

In the present study, we demonstrate for the first time that intravenous administration of xenogeneic hAT-MSCs can alleviate Df-induced murine AD, presumably through the regulation of B cell-mediated IgE production. Our findings are consistent with the previous reports from several groups although the sources for MSC isolation are different. Na et al. reported that bone marrow-derived MSCs ameliorated ovalbumin-induced AD model by inhibiting B cell responses [27]. In our previous study, hUCB-MSCs alleviated Df-induced AD model through the regulation of mast cell function [14]. Interestingly, in the study, although sufficient effects could be obtained in both intravenous and subcutaneous delivery of MSCs, the potency of serum IgE down-regulation was significantly higher in intravenously injected group. Consistently, in the present study, the i.v. administration of hAT-MSCs resulted in the significant decrease in IgE level. To address this discrepancy between two delivery routes, we performed the sensitive assay to track cell distribution. In the previous study, hUCB-MSCs were infused after labeling with fluorescence to track the cells. While s.c. injected hUCB-MSCs were detected around the skin lesion as clumped cells at day 3, i.v. injected cells were hardly detectable at the same day. Since fluorescence-mediated cell tracking is known to have relatively low sensitivity and specificity, especially when a few cells are distributed, real time qPCR was conducted for minute detection of human cells and further quantification. At two hours and three days after hAT-MSC administration, the majority of cells were detected in the lung and heart. Surprisingly, cells were undetectable at week 2 after administration. It seems that short time of cell distribution in the body less than 2 weeks is sufficient to alter the systemic immune network. Moreover, because MCs are degranulated after local infiltration around the lesion, decrease in MC degranulation might be the subsequent result of IgE regulation by hAT-MSCs. A further study investigating MSC distribution with frequent time interval would reveal more precise pattern of cell migration and therapeutic mechanisms involving target immune cells.

Recently, several studies have raised the concerns regarding the intravenous application of cell therapeutics including MSCs. The main concern is that the majority of intravenously infused cells are harbored in the lung, resulting in the pulmonary embolism [28, 29]. In the present study, 8 mice administered with 2 × 10^5^ cells and 47 mice injected with 2 × 10^6^ cells did not exhibit any embolism-related symptoms, supporting the safety of hAT-MSCs. Besides, the embolism-mediated lethality has been reported to be caused by the rapid velocity of cell administration rather than the toxicity of cell itself.

The immunomodulatory effect of MSCs on immune cells is exerted by soluble factors, such as prostaglandin-E_2_ (PGE_2_), IDO-1, NO, TGF-β1, hepatocyte growth factor (HGF), and interleukin (IL)-10 [3, 30–35]. Among several factors, we have proven that COX-2-synthesized PGE_2_ is pivotal in therapeutic effects of hMSCs against immune-related diseases, including inflammatory bowel disease and atopic dermatitis [14, 36–38]. In addition, recent studies from other groups showing the suppressive effect of MSCs on AD-related immune cells emphasized the role of COX-2- PGE_2_-dependent mechanisms [12, 39]. More specifically, a large body of studies has focused on demonstrating the interaction between MSCs and B cells [1, 40–42]. Corcione et al. reported that soluble factors released from MSCs exert a suppressive effect on the B cell proliferation and differentiation [40]. Another study from Asari et al. supported this finding by showing that humoral factors from MSCs inhibit terminal differentiation of B cells [1]. Consistent with these reports, in the present study, hAT-MSCs not only decreased the proliferation of B cells but prevented the maturation of B cells to down-regulate IgE production. Moreover, Su et al. proved that secreted factors from MSCs attenuated allergic conjunctivitis through COX-2-dependent multiple antiallergic mechanisms including B cell inhibition [42]. In agreement with the finding, we found here that hAT-MSCs exploit the COX-2 signaling to suppress both proliferation and maturation of B cells. In a more mechanistic approach, Franquesa et al. recently demonstrated that hAT-MSCs abrogate plasmablast formation in T cell-dependent manner and induce regulatory B cells independently of T cells [41]. In our study, hAT-MSCs directly inhibited the proliferation and maturation of B cells via COX-2 signaling. This discrepancy in B cell regulation by T cells might be resulted from the differences in the sources of B cell or MSC isolation as well as co-culture conditions. Since it has been recently reported that diverse subsets of helper T cells such as Th1, Th17 and Th22, as well as classically dominant Th2, might be involved in the pathogenesis of AD [43, 44], future studies are required to uncover novel mechanisms of hMSC action on other subsets of immune responses including various helper T cell subtypes as well as dendritic cell, B cell, mast cell and basophil.

One might envision that soluble factors from MSCs can be refined as lotion type and topically applied for the treatment of AD, because lotion type drugs moisturized with anti-inflammatory or anti-itch activity are ideal as well as encouraged. However, the administration of MSC itself has advantages in that the efficacy of topically applied anti-inflammatory or –itch agents can be limited due to low efficiency of transdermal delivery and that MSC administration can reconstitute the immune network through the production of multiple immunomodulatory factors in response to inflammatory stimuli, elevated in disease environment. In our previous study, we showed that MSCs produce higher level of TGF-β1 in response to IL-4, a dominant cytokine in AD environment, resulting in the suppression of mast cell degranulation [14].

In conclusion, our present study revealed that the intravenous administration of hAT-MSCs might be a promising therapeutic alternative for AD, and that COX-2 signaling is crucial factor for regulation of B cell maturation as well as MC degranulation.

## MATERIALS AND METHODS

### Isolation and culture of hAT-derived MSCs

hAT-MSCs were isolated from freshly excised human fat tissue obtained from the waste after liposuction. Fat tissue was digested with 0.1% collagenase (type I, Gibco) for 30 minutes at 37°C after washing with α-MEM (Gibco, Grand Island, NY), The pellet was obtained by centrifugation at 2,000 rpm for 10 min followed by the filtration with 100 μm nylon mesh. Suspended cells were incubated in α-MEM containing 8% fetal bovine serum at 37°C with 5% CO_2_. All procedures using human adipose tissue or adipose tissue-derived cells were conducted in accordance with guidelines approved by institutional review board (IRB No. P01-201511-31-004). For this study, hAT-MSCs isolated from five different donors were used after verification of characteristics for MSCs by observing the surface markers and differentiation capabilities (Supplementary Table S1 and Supplementary Figure S1A–S1C in Supplementary Data).

### Reagents

Df extract was purchased from Biostir Inc. (Hiroshima, Japan). N-nitro-L-arginine methyl ester (L-NAME), 1-methyl-tryptophan (1-MT) and celecoxib were purchased from Sigma-Aldrich (St. Louise, MO).

### Mice

NC/Nga mice (male, 8wk old) were obtained from SLC (Hamamatsu, Japan) and group housed under specific pathogenic-free conditions in the animal facility of the Seoul National University. All experiments were approved by and followed the regulations of the Institute of Laboratory Animal Resources (SNU-140320-1), Seoul National University, South Korea.

### Atopic dermatitis model induction in NC/Nga mice

Atopic dermatitis-like symptoms were induced according to the previously described method [45, 46]. Briefly, the upper backs of the mice were shaved with a clipper. Sodium dodecyl sulfate (4%, 150 μL/head) was treated on the shaved dorsal skin including surfaces of ears to achieve skin barrier disruption. After 3–4 hours, 100 mg of Df extract (Biostir Inc., Hiroshima, Japan) was topically applied. Df extract was treated twice a week for three weeks (total six times). hAT-MSCs (2 × 10^5^ or 2 × 10^6^ cells/200 μL normal saline) were injected intravenously on day 21 (Figure 1A). The sum of the individual scores (0, none; 1, mild; 2, moderate; 3, severe) on dryness, excoriation, erythema and edema was calculated to determine the clinical severity. After sacrifice on day 35, serum and skin samples were collected to detect the concentration of total IgE using a commercial ELISA kit (BD Bioscience, San Jose, CA) or to evaluate histopathological lesions, respectively.

### Histopathological evaluation

Skin samples were collected, fixed in 10% formalin followed by consecutive tissue processing steps including alcohol-xylene changes, and embedding in paraffin. Sections of 5 μm thickness were prepared and stained with H&E or toluidine blue. Leukocyte infiltration was determined by H&E staining. Mast cell infiltration and degranulation were measured by toluidine blue staining.

### Tracking of cell distribution

To trace the distribution of injected cells, hMSC concentration in major organs was analyzed by real-time qPCR with human Alu primers. hAT-MSCs (2 × 10^6^ cells in 200μL normal saline) were injected intravenously into dermatitis-induced mice. At 2 hours, 3 days, 2 weeks and 4 weeks after cell injection, mice were sacrificed followed by DNA isolation from kidney, heart, lung, skin, brain, blood, lymph node, testis, bone marrow, spleen, pancreas and liver. Standard samples of mouse skin DNA containing 16%, 8%, 4%, 2%, 1%, 0.5%, 0.25%, 0.125%, 0.062%, 0.031%, 0.016%, 0.008%, 0.004% and 0% (negative control) of hAT-MSC DNA were prepared and *Cp* values were determined respectively to obtain standard curves. The detection limit was fixed as 0.008% because *Cp* value of 0.008% hAT-MSCs exhibited significant difference from that of negative control. Based on the standard curve, the concentration of hMSCs in each sample was calculated. The sequences of Alu primers used in real-time qPCR are as follows; GTCAGGAGATCGAGACCATCCC (forward) and TCCTGCCTCAGCCTCCCAAG (reverse).

### B cell isolation and analysis

Human B lymphocytes were isolated from human umbilical cord blood. Cord blood samples were obtained immediately after delivery, and the informed consent of the mother was given and approved by the Boramae Hospital Institutional Review Board (IRB) and the Seoul National University IRB (IRB No. 1109/001-006). The cord blood samples were mixed with Hetasep solution (StemCell Technologies, Vancouver, Canada) and incubated for more than 1 hour at room temperature to remove erythrocytes. After Ficoll density-gradient centrifugation at 2,500 rpm for 20 min, mononuclear cells were isolated. B cells were negatively isolated from mononuclear cells using naïve B cell isolation kit according to the manufacturer's instruction (Miltenyi Biotec, San Diego, CA). B cell maturation was induced with 100 ng/mL CD154 (CD40 ligand) and 25 ng/mL interleukin (IL)-4 for 5 days. For co-culture experiments of B cells with hAT-MSCs, B cells were cultured with hAT-MSCs after the induction of B cell maturation for additional 5 days. The proliferation of B cell was measured by counting the number of CD19-positive cells using flow cytometry (BD Biosciences, San Jose, CA). B cell maturation was determined by the analysis of surface or intracellular marker using flow cytometry. For surface marker staining, B cells were fixed and incubated with PerCP conjugated anti-CD19, PE conjugated anti-CD27 and FITC conjugated anti-CD38. For intracellular marker staining, cells were fixed and permeabilized with an intracellular staining buffer set (BD Biosciences) and then incubated with FITC conjugated anti-IgE antibody after Fc receptor (FcR) blocking using FcR blocking reagent (Miltenyi Biotec, Bergisch Gladbach, Germany). All flow cytometry analyses were performed on a FACS Calibur and the Cell Quest software (BD Biosciences).

### Mast cell culture

The human mast cell line LAD2, developed from the bone marrow of a patient with mast cell leukemia, was kindly provided by Professor Dr. D. D. Metcalfe of the Center for Cancer Research, National Institutes of Health (Bethesda, MD). These cells were cultured in StemPro-34 serum-free medium (SFM) supplemented with 2 mM l -glutamine, 100 IU/mL penicillin, 50 μg/mL streptomycin and 100 ng/mL recombinant human stem-cell factor (rhSCF), as described previously [47]. Cell lines were tested and authenticated by measuring the expression of surface markers including CD117 and FcεRI every two weeks by flow cytometry.

### Mast cell degranulation

To induce mast cell degranulation, LAD-2 cells were sensitized with 100 ng/mL human myeloma IgE (Millipore, Billerica, MA) for 24 h. Cells that were re-suspended at 1 × 10^6^ cells/mL were challenged with 10 μg/mL anti-IgE (Millipore). hAT-MSCs were added when anti-IgE was challenged. After 1 hour, degranulation was stopped by replacing the plates on ice and supernatants were harvested after centrifugation. β-hexosaminidase (β-hex) release in supernatant was detected to assess the degranulation. Supernatant (60 μL) was transferred to a 96-well plate and an equal volume of substrate solution (7.5 mM ρ-nitrophenyl-Ν-acetyl-β-D-glucosaminide dissolved in 80 mM citric acid, pH 4.5) was added. The mixture was incubated on a shaking incubator for 90 minutes at 37°C followed by the addition of 120 μL of 0.2 M glycine (pH 10.7). The absorbance was measured at 450 nm using a microplate reader (TECAN, Zürich, Switzerland). β–hex release was calculated as the percentage of total β-hex content in naive mast cells, measured after cell lysis with 0.1 % Triton X-100.

### Statistical analysis

The mean values of the different groups were expressed as the mean ± SD. All statistical comparisons were made using one-way ANOVA followed by the Bonferroni post-hoc test for multi-group comparisons using the GraphPad Prism version 5.01 (GraphPad Software, San Diego, CA). Statistical significance designated as asterisks is indicated in the figure legends.

## SUPPLEMENTARY MATERIALS


