# Supplementary material for: Human adipose tissue-derived mesenchymal stem cells alleviate atopic dermatitis via regulation of B lymphocyte maturation
Source: Oncotarget. 2016 Nov 19;8(1):512–22. doi: 10.18632/oncotarget.13473 (PMC5352174; doi:10.18632/oncotarget.13473)
Supplement: Supplementary file 1 [file oncotarget-08-512-s001.pdf]

Human adipose tissue-derived mesenchymal stem cells alleviate atopic dermatitis via regulation of B lymphocyte maturation

Supplementary Materials

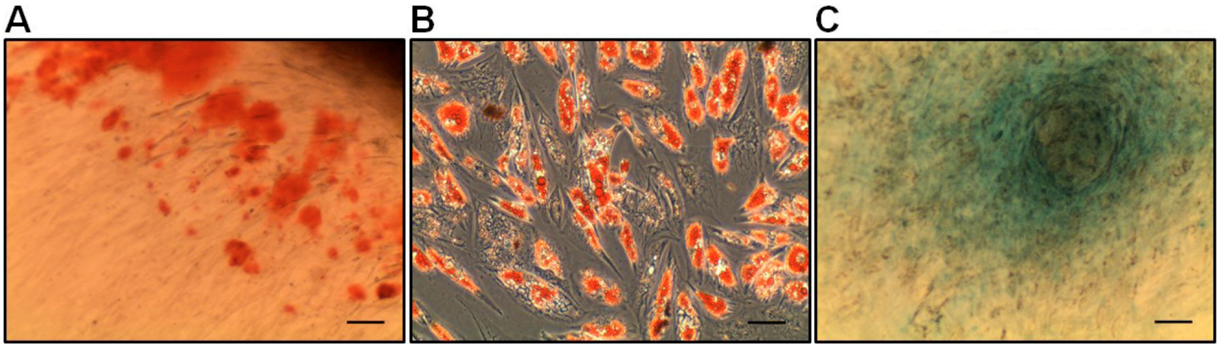

Supplementary Figure S1: Differentiation of hAT-MSCs into osteocytes, adipocytes and chondrocytes. hAT-MSCs were induced to differentiate into specific cell types of mesenchymal lineage. (**A**) Osteogenic differentiation was induced and calcium deposits were stained with Alizarin Red S. (**B**) Adipogenic differentiation was induced and lipid droplet accumulation was visualized with Oil Red O staining. (**C**) Chondrogenic differentiation was induced and proteoglycan synthesis by chondrocytes was detected by Alcian Blue staining, scale bar = 500 µm.

Characterization of human adipose tissue-derived mesenchymal stem cells (hAT-MSCs)

Flow cytometric analysis

hAT-MSCs isolated from 5 different donors were stained with fluorescense-conjugated antibodies specific for human CD14, CD45, CD73, CD90, CD105. Cells stained with non-specific isotype-matched antibodies were served as controls. Antibodies were purchased from BD Bioscience (San Jose, CA), and flow cytometric analysis was performed on a FACSCaliber using the Cell Quest software (BD Biosciences).

Osteogenic differentiation

AT-MSCs were induced to differentiate into osteocytes using StemPro® Osteogenesis Differentiation Kit (Gibco, Grand Island, NY) according to manufacturer's protocol. Briefly, cells (1×10^5 /well) were seeded in 6-well plates, and 3 days after seeding the cells were replaced with differentiation medium. The cells were grown for 3 weeks, with medium replacement twice a week. Osteogenesis was detected by Alizarin Red staining. The cells were fixed with 4% paraformaldehyde followed by the staining with Alizarin Red S solution for 10 minutes at room temperature. Images of stained cells were captured using light microscope.

Adipogenic differentiation

hAT-MSCs were induced to differentiate into adipocytes using StemPro® Adipogenesis Differentiation Kit (Gibco) according to manufacturer's protocol. After 21 days or longer, adipogenesis was determined by Oil Red O staining. The cells were fixed and stained with Oil Red O solution for 2 hours at room temperature. Images of stained cells were captured using light microscope.

Chondrogenic differentiation

hAT-MSCs were induced to differentiate into chondrocytes using StemPro® Chondrogenesis Differentiation Kit (Gibco) according to manufacturer's protocol. After 3 weeks, chondrogenesis was assessed by Alcian Blue staining. The cells were fixed and stained with Alcian Blue solution for 30 minutes at room temperature. Images of stained cells were captured using light microscope.

Supplementary Table S1: Analysis of surface marker expression in hAT-MSCs

Lot #	% of cells				
	$CD34^+$	$CD45^+$	$CD73^+$	$CD90^+$	$CD105^+$
EHL-AD-E-0001	0.58	0.19	99.75	99.75	99.92
EHL-AD-E-0002	1.02	1.56	99.59	99.89	99.88
EHL-AD-E-0003	1.33	2.14	99.51	99.72	98.17
EHL-AD-E-0004	1.55	1.27	99.29	99.85	97.87
EHL-AD-D-0001	0.64	0.41	99.59	99.76	99.29
